# Tanshinone IIA and Cryptotanshinone Prevent Mitochondrial Dysfunction in Hypoxia-Induced H9c2 Cells: Association to Mitochondrial ROS, Intracellular Nitric Oxide, and Calcium Levels

**DOI:** 10.1155/2013/610694

**Published:** 2013-03-04

**Authors:** Hyou-Ju Jin, Chun-Guang Li

**Affiliations:** ^1^Traditional & Complementary Medicine Program, RMIT Health Innovations Research Institute, School of Health Sciences, RMIT University, Bundoora, VIC 3083, Australia; ^2^Center for Complementary Medicine Research, National Institute of Complementary Medicine, University of Western Sydney, Campbelltown Campus, Penrith, NSW 2751, Australia

## Abstract

The protective actions of tanshinones on hypoxia-induced cell damages have been reported, although the mechanisms have not been fully elucidated. Given the importance of nitric oxide (NO) and reactive oxygen species (ROS) in regulation of cell functions, the present study investigated the effects of two major tanshinones, Tanshinone IIA (TIIA) and cryptotanshinone (CT), on hypoxia-induced myocardial cell injury and its relationships with intracellular NO and ROS, calcium, and ATP levels in H9c2 cells. Chronic hypoxia significantly reduced cell viability which accompanied with LDH release, increase in mitochondrial ROS, intracellular NO and calcium levels, decrease in superoxide dismutase (SOD) activity, and cellular ATP contents. TIIA and CT significantly prevented cell injury by increasing cell viability and decreasing LDH release. The protective effects of tanshinones were associated with reduced mitochondrial superoxide production and enhanced mitochondrial SOD activity. Tanshinones significantly reduced intracellular NO and Ca^2+^ levels. ATP levels were also restored by TIIA. These findings suggest that the cytoprotective actions of tanshinones may involve regulation of intracellular NO, Ca^2+^, ATP productions, mitochondrial superoxide production, and SOD activity, which contribute to their actions against hypoxia injuries.

## 1. Introduction

It has been established that chronic hypoxia is associated with cardiac dysfunctions in certain pathological conditions such as ischemia reperfusion, myocardial infarction (MI), and hypertrophy [1]. Hypoxia causes changes of various cellular mechanisms related to mitochondrial dysfunction and oxidative stress [2]. Among these, hypoxia-induced changes of ROS and NO productions, intracellular calcium, and ATP levels may have particular importance, given the role of these molecules in regulation of cell functions in general [3]. For example, a recent study shows that hypoxia-increased mitochondrial superoxide anion (O_2_^∙−^), not cytosolic O_2_^∙−^, plays an important role in hypoxia-induced cell apoptosis [4, 5]. Studies have also found that excess NO production by hypoxia can result in mitochondrial ROS increase by inhibiting mitochondrial electron transport chain function, which in turn promotes peroxynitrite formation and cell apoptosis [6, 7]. On the other hand, hypoxia may modulate NO production by regulating intracellular calcium which is important for Ca^2+^/calmodulin-dependent eNOS and nNOS activity, and NO increase in turn may inhibit mitochondrial complex IV [8]. This indicates an interaction among NO, ROS, intracellular calcium, and regulation of ATP synthesis in mitochondria. Understanding the relationship of these factors may help to interpret the mechanisms of cellular injury in hypoxia condition [9, 10].

Tanshinones are a group of bioactive compounds isolated from *Salvia miltiorrhiza* (Danshen), a traditionally medicinal plant used in management of angina pectoris, atherosclerosis, and MI [11]. Among these, tanshinone IIA (TIIA) and cryptotanshinone (CT) are two major bioactive tanshinones [12]. They have been reported to have actions against oxidative stress, myocardial infarction, and myocardial ischemia reperfusion injury [13]. For example, studies *in vitro* have revealed antioxidant actions of TIIA by attenuating intracellular ROS level and enhancing antioxidant enzymes activity [14, 15]. TIIA and CT have also been shown to influence vasodilation by regulating NO and intracellular Ca^2+^ levels in endothelial cells [16, 17]. However, the actions of TIIA and CT on ROS and NO pathways under hypoxic conditions are still not clear. Thus, the present study was conducted to investigate the effects of TIIA and CT on hypoxia-induced cardiac injury and their regulations of intracellular NO, ROS, calcium levels, and ATP contents in H9c2 cells.

## 2. Materials and Methods

### 2.1. Chemicals

Tansinone IIA (TIIA) and cryptotanshinone (CT) were purchased from the National Institute for the Control of Pharmaceutical and Biological Products (>99% purity) (Beijing, China). Dulbecco's Modified Eagle's Medium (DMEM), fetal bovine serum (FBS), penicillin, and streptomycin were purchased from Gibco BRL (Grand Island, NY, USA). GasPak EZ Anaerobe Container System Sachets with Indicator and GasPak EZ Standard Incubation Container were from Becton Dickinson and company (Sydney, NSW, Australia). Trypsin-EDTA solution, (3-(4,5-dimethylthiazol-2-yl)-2,5-diphenyltetrazolium bromide), 2′,7′-dichlorodihydrofluorescein diacetate, Superoxide dismutase assay kit, dihydroethidium, diphenyleneiodonium chloride, 4-hydroxy-TEMPO (TEMPOL), rotenone, antimycin A and nitro-L-arginine methyl ester (L-NAME) were from Sigma-Aldrich (St. Louis, MO, USA). Fura-2 AM and MitoSOX were from Molecular Probes (S. San Francisco, CA, USA). Lucigenin and MnTBAP were from Santa Cruz Biotechnology (CA, USA). CytoTox96 NonRadioactive Cytotoxicity assay kit and ENLITEN ATP Assay System Bioluminescence Detection Kit were from Promega (Madison, WI, USA). 4, 5-Diaminofluorescein (DAF-2) was purchased from Sapphire Bioscience Biochemicals (Sydney, NSW, Australia). Mitochondria Isolation Kit for Cultured Cells was purchased from Thermo Scientific (Rockford, USA).

### 2.2. Cells Culture and Hypoxia

The H9c2 embryonic rat heart-derived the cells were obtained from American Type Culture Collection (ATCC; Manassas, VA) and maintained in Dulbecco's modified Eagle's medium supplemented with 10% v/v fetal bovine serum and 100 **μ**g/mL penicillin/streptomycin at 37°C in a humidified atmosphere containing 5% CO_2_ (passage 25–35).

To mimic hypoxia condition, cells were placed in a GasPak EZ Gas generating Pouch System (Becton-Dickinson) for 8 hr and incubated with serum-free and glucose-free DMEM as described previously [18]. As normoxia control, serum-free DMEM was added to cells and incubated for 8 hr in normoxia condition (21% O_2_). For the treatment groups, TIIA or CT (3 *μ*M) was added 2 hr before and during the hypoxia period. The experimental condition was established from a preliminary study involving different concentrations of tanshinones (0.1–10 *μ*M) at different (2 and 24 hr) pre- and posthypoxia incubation periods. In some experiments, MnTBAP (1 **μ**M), rotenone (10 *μ*M), antimycin A (AA: 10 *μ*M), TEMPOL (10 mM), and L-NAME (1 mM) were treated 1 hr before inducing hypoxia as positive controls.

### 2.3. MTT Assay

Cell viability was determined by MTT (3-(4,5-dimethylthiazol-2-yl)-2,5-diphenyltetrazolium bromide) assay as described previously with a modification [19]. The cells (1 × 10^4^ cells/well) were seed in 96 wells. At the end of hypoxia period, MTT solution was added into plates at a final concentration of 0.5 mg/mL and incubated for 2 hr at 37°C. Then, the culture medium was discarded and 150 *μ*L DMSO was added to each well to dissolve dark blue formazan crystals. The absorbance was read at 570 nm using POLARstar OPTIMA microplate reader (BMG LabTech).

### 2.4. LDH Release Measurement

LDH release was determined by CytoTox 96 NonRadioactive Cytotoxicity Assay kit according to the manufacturer's instructions (Promega). After 8 hr hypoxia, the supernatant was collected and placed in 96 wells and 50 *μ*L of reconstitute substrate mixture was added in each well. After 30 mins incubation, 50 *μ*L of stop solution was added and absorbance was measured at 490 nm using Flexstation multiplate reader (Molecular Devices). 

### 2.5. Cellular ATP Content Measurement

Cellular ATP content was measured by ENLITEN ATP Assay System Bioluminescence Detection Kit according to the manufacturer's instructions (Promega). After hypoxia, The cells were washed with PBS and lysated, and supernatants were collected. Proteins (10 *μ*g/20 *μ*L) were added in white optiplate and initiated action by adding reconstituted reagent. Then, luminescence was measured in POLARstar OPTIMA microplate reader (BMG LabTech).

### 2.6. NADPH Oxidase Activity

NADPH oxidase activity was measured by lucigenin chemiluminescence as described previously with minor modification [20]. After 8 hr hypoxia, the cells were collected and centrifuged at 750 g for 10 mins at 4°C. The supernatant was discarded and the pellet was resuspended in lysis buffer (50 mM KH_2_PO_4_, pH 7.0, 1 mM EGTA, 10 *μ*g/mL aprotinin, 0.5 *μ*g/mL leupeptin, 1 *μ*g/mL pepstatin, and 0.5 mM PMSF). Next, the cells were homogenized by quick freeze and thaw step. 10 *μ*g of proteins were added in optiwhite 96-well plates with the 100 *μ*L assay buffer (50 mM KH_2_PO_4_ (pH 7.0), 150 mM Sucrose, 100 **μ**M NADPH, and 1 mM EGTA). Then, the reaction was started by 5 *μ*M lucigenin. 5 *μ*M DPI was added as an inhibitor. Chemiluminescence was measured with POLARstar OPTIMA microplate reader (BMG LabTech). 

### 2.7. Intracellular Hydrogen Peroxide/Peroxynitrite Production

Intracellular hydrogen peroxide/peroxynitrite (H_2_O_2_/ONOO^−^) production was measured by using 2′,7′-dichlorofluoresceindiacetate (DCFH-DA). The nonfluorescent DCFH-DA readily diffuses into the cells, where it is hydrolysed to the polar derivative DCFH, which is oxidized in the presence of H_2_O_2_ or ONOO^−^ to the highly fluorescent DCF. At the End of hypoxia, the cells were incubated with 20 *μ*M DCFH-DA for 20 mins at 37°C. The fluorescence was measured at an excitation wavelength of 488 nm and emission at 530 nm in Flexstation multiplate reader (Molecular Devices). The fluorescence intensity was normalized to total protein contents and expressed as arbitrary units per mg protein.

### 2.8. Intracellular and Mitochondrial Superoxide Production

Intracellular and mitochondrial superoxide production was measured by loading cells with 20 *μ*M dihydroethidium (DHE) and 2 *μ*M MitoSOX, respectively, by following method described previously with minor modification [21]. End of hypoxia, DHE and MitoSOX were added and incubated for 30 mins at 37°C including 10 mins DAPI (1 *μ*M) staining. After the incubation, the cells were washed with PBS. The cell images were obtained using Image Xpress MICRO system (Molecular Devices) at 20X magnification with binning of 1 and gain of 2 using laser-based focusing. Images were captured using a DAPI filter (350/70 nm Ex, 470/50 nm Em for DAPI) and Cy3 filter (550/35 nm Ex, 570/30 nm Em for DHE and MitoSOX). The cell images were analysed by Meta express software (Molecular Devices).

### 2.9. Superoxide Dismutase Activity

Superoxide dismutase activity was determined by superoxide dismutase assay kit according to the manufacturer's instructions (Sigma-Aldrich). Briefly, cytosolic and mitochondria fractions were prepared by Mitochondria Isolation kit (Thermofisher). The protein amount was measured by Bradford assay. 20 *μ*g of protein was added in 96-well plates and then the reaction was initiated by adding enzyme solution. Absorbance was measured at 450 nm with POLARstar OPTIMA microplate reader (BMG LabTech). 

### 2.10. Intracellular Nitric Oxide Production

Direct measurement of intracellular nitric oxide production was performed by loading 4,5-diaminofluorescein (DAF-2) [22]. The cells were incubated with 5 *μ*M DAF-2 for 1 hr after hypoxia. Following the incubation, the cells were washed twice with PBS and visualized in Image Xpress MICRO system (Molecular Devices) at 20X magnification with binning of 1 and gain of 2 using laser-based focusing. Images were captured using a DAPI filter (350/70 nm Ex, 470/50 nm Em for DAPI) and GFP filter (490/40 nm Ex, 510/50 nm Em for DAF-2). The cell images were analysed by Meta Express software (Molecular Devices).

### 2.11. Intracellular Calcium Level Measurement

Intracellular calcium level was determined by Fura-2AM as described previously with minor modification [23]. After hypoxia, the cells were rinsed twice with PBS and detached by trypsinization. The detached cells were centrifuged and washed with PBS once. Then, the cells were incubated with HBSS with Ca^2+^ buffer (140 mM NaCl, 4.2 mM KCl, 1 mM CaCl_2_, 0.4 mM MgSO_4_, 0.4 mM Na_2_HPO_4_, 0.5 mM NaH_2_PO_4_, 0.3 mM MgCl_2_, 5 mM glucose, and 0.2% bovine serum albumin, pH 7.4) supplemented with 2 *μ*M Fura-2AM for 30 mins at 37°C. After the incubation, the cells were resuspended to HBSS buffer only and incubated for 30 mins at room temperature. 1 × 10^5^ cells were transferred to 96-well plates, and then Fura-2AM fluorescence was obtained by alternate excitation at 340 and 380 nm and the emission was detected at 510 nm. The fluorescence maximum was determined by lysing cells with 0.2% Triton X-100 and fluorescence minimum was obtained by recording fluorescence following addition of 40 mM EDTA. The calcium concentration was calculated by equation according to what previously described [24].

### 2.12. Statistical Analysis

Results were expressed as means ± SEM. Statistical differences among groups were analysed by one-way analysis of variance (ANOVA) using GraphPad Prism Software version 5.0. *P* ≤ .05 was considered significant. 

## 3. Results

### 3.1. Effects of TIIA and CT on Hypoxia-Induced Cell Injury

**Figure 1:**
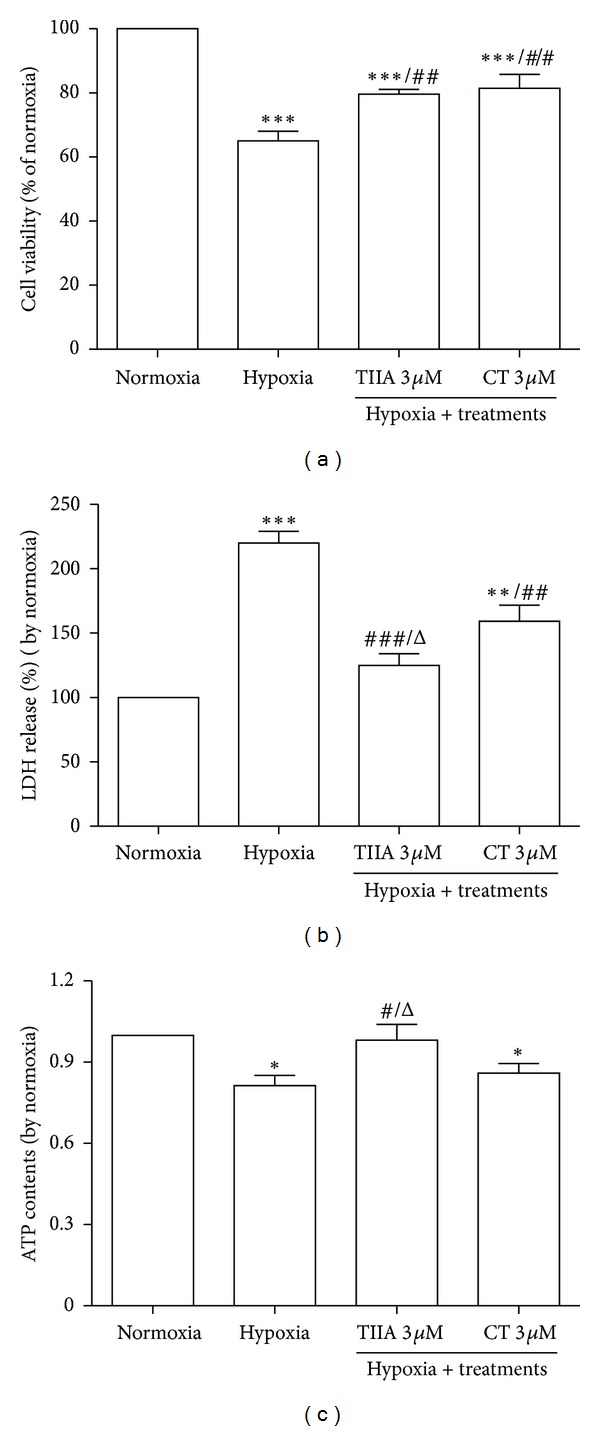
Effects of TIIA and CT on hypoxia-induced H9c2 cell injury. (a) Cell viability by MTT assay. The cell viability of normoxia was adjusted to 100% (*n* = 5), ^##^ *P* < 0.01  *versus *hypoxia, ***P < 0.001  *versus* normoxia. (b) LDH release. The LDH release of normoxia was adjusted to 100% (*n* = 3), Δ*P* < 0.05  *versus CT,* ^##^ *P* < 0.01  *versus* hypoxia, and ^###^ *P* < 0.001  *versus* hypoxia, ***P < 0.001 *versus* normoxia. (c) Cellular ATP contents. The cellular ATP contents of normoxia was adjusted to 1 (*n* = 6), *P < 0.05  *versus *normoxia, *P < 0.05  *versus* normoxia, ^#^ *P* < 0.05  *versus* hypoxia, and  ^Δ^ *P* < 0.05  *versus CT*.

Cells exposed to a 8 hr hypoxia exhibited a significant decrease in cell viability (*P* < 0.001), measured by MTT assay, which was significantly inhibited by pretreatment of TIIA and CT (3 *μ*M) (*P* < 0.01) (Figure 1(a)). 8 hr hypoxia also significantly increased LDH release (to 220.0%, *P* < 0.001), which was significantly inhibited by 3 *μ*M TIIA and CT (*P* < 0.001). The value of LDH release in TIIA-treated group was significantly less than that of LDH release in CT group (*P* < 0.05) (Figure 1(b)). After 8 hr hypoxia, a significant reduction of the cellular ATP contents (by 20.9%, *P* < 0.05) was observed compared to normoxia control, which was restored by pretreatment with TIIA. The cellular ATP contents in TIIA-treated group were significantly higher (*P* < 0.05) than those in CT group (Figure 1(c)). 

### 3.2. Effects of TIIA and CT on Hypoxia-Induced Decrease in Intracellular Superoxide Generation and NADPH Oxidase Activity

**Figure 2:**
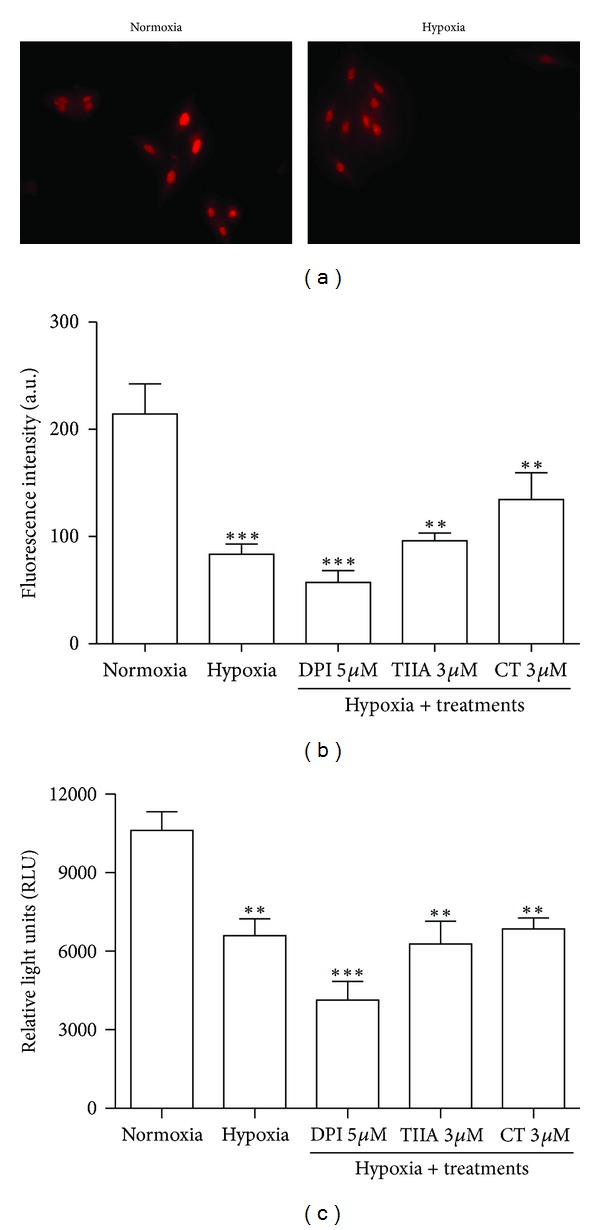
Effects of TIIA and CT on hypoxia-induced decrease in intracellular superoxide level and NADPH oxidase activity. (a) Images of cells labelled with DHE in normoxia and hypoxia groups. (b) The quantified value of DHE fluorescence intensity. Represented data are mean value of 500 each cells with 4 independent experiments. (c) Quantitative value of NADPH oxidase activity. Data shown are representative of 4 independent experiments.  **P < 0.01  *versus normoxia*,  ***P < 0.001  *versus normoxia. *

8 hr hypoxia significantly decreased intracellular superoxide generation (*P* < 0.001) and NADPH oxidase activity (*P* < 0.01) compared to normoxia control. TIIA-, CT- and DPI-treated groups did not significantly affect the intracellular superoxide generation and NADPH oxidase activity compared to hypoxia control (Figures 2(b) and 2(c)). 

### 3.3. Effects of TIIA and CT on Hypoxia-Induced Increase in H_2_O_2_/ONOO^−^ Production

**Figure 3:**
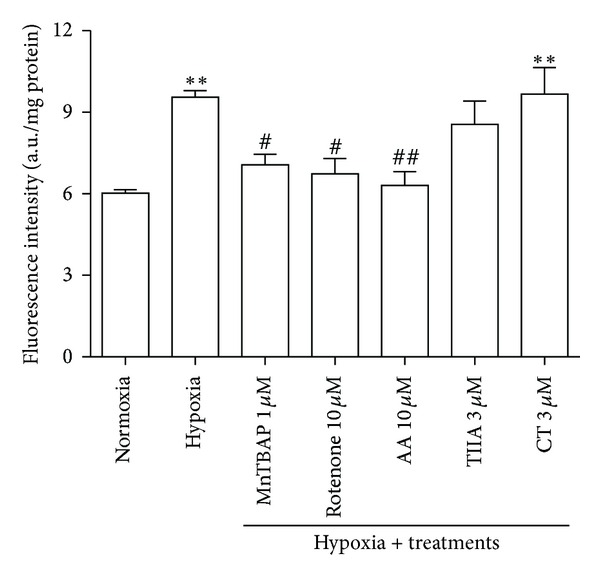
Effects of TIIA and CT on hypoxia-induced increase in H_2_O_2_/ONOO^−^ production. Quantitative value of DCFH-DA fluorescence intensity (*n* = 4). ^#^ *P* < 0.05  *versus hypoxia,* ^##^ *P* < 0.01  *versus hypoxia*, and **P < 0.01* versus normoxia. *

After 8 hr hypoxia, the DCF fluorescence intensity was significantly elevated to 9.57 ± 0.50 a.u/mg protein (*P* < 0.01), presenting 59% increase compared to normoxia group. The hypoxia-induced increase in DCF fluorescence was abolished by 1 *μ*M MnTBAP (peroxynitrite inhibitor), 10 *μ*M rotenone (complex I inhibitor), and antimycin A (AA) (complex III inhibitor), presenting 70.1%, 79.2%, and 91.4% inhibition rates, respectively. Also, 3 *μ*M TIIA reduced the increase in DCF fluorescence by 28.9% without statistical significance difference compared to hypoxia control (Figure 3). 

### 3.4. Effects of TIIA and CT on Hypoxia-Induced Increase in Mitochondrial Superoxide Production

**Figure 4:**
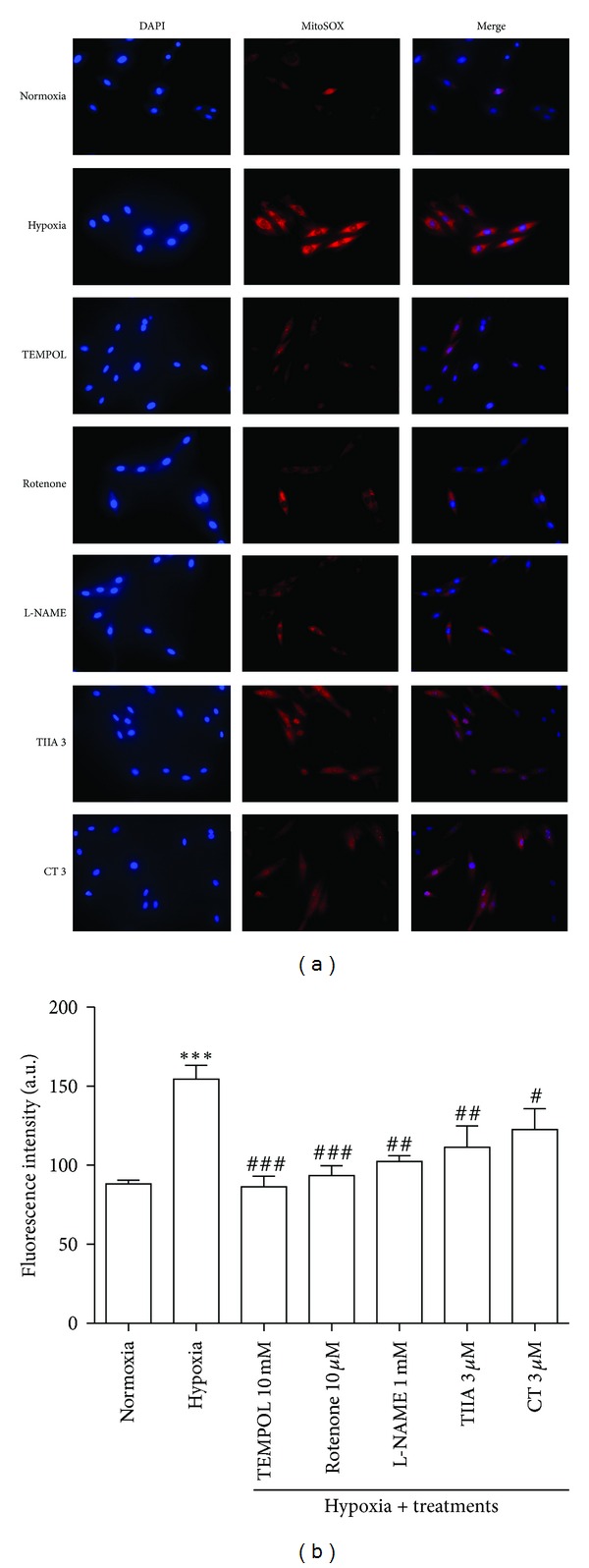
Effects of TIIA and CT on hypoxia-induced increase in mitochondrial superoxide production. (a) Cell images illustrate MitoSOX (red) and DAPI (blue) in each group. (b) The quantified value of MitoSOX fluorescence intensity. Represented data are mean value of 500 each cells with 5 independent experiments. ^#^ *P* < 0.05  *versus* hypoxia, ^##^ *P* < 0.01  *versus* hypoxia, ^###^ *P* < 0.001  *versus* hypoxia, and ***P < 0.001 versus normoxia.

Figure 4(a) illustrates the fluorescence images of cells stained with MitoSOX. When compared with normoxia group, distinct intensification in fluorescence was observed in hypoxia group. The cells exposed to hypoxia significantly increased MitoSOX fluorescence intensity to 154.3 ± 19.9 arbitrary units (a.u) while normoxia showed 88.2 ± 5.3 a.u. 1 hr pretreatment with 10 mM TEMPOL (SOD mimic), 10 *μ*M rotenone (complex I inhibitor), and 1 mM L-NAME (NO synthase inhibitor) significantly decreased the mitochondrial superoxide production. In the presence of TIIA and CT (3 *μ*M), the mitochondrial superoxide production was significantly reduced to 111.4 ± 30.2 a.u (*P* < 0.01) and 122.5 ± 29.7 a.u (*P* < 0.05), respectively (Figure 4(b)). 

### 3.5. Effects of TIIA and CT on SOD Activity

**Figure 5:**
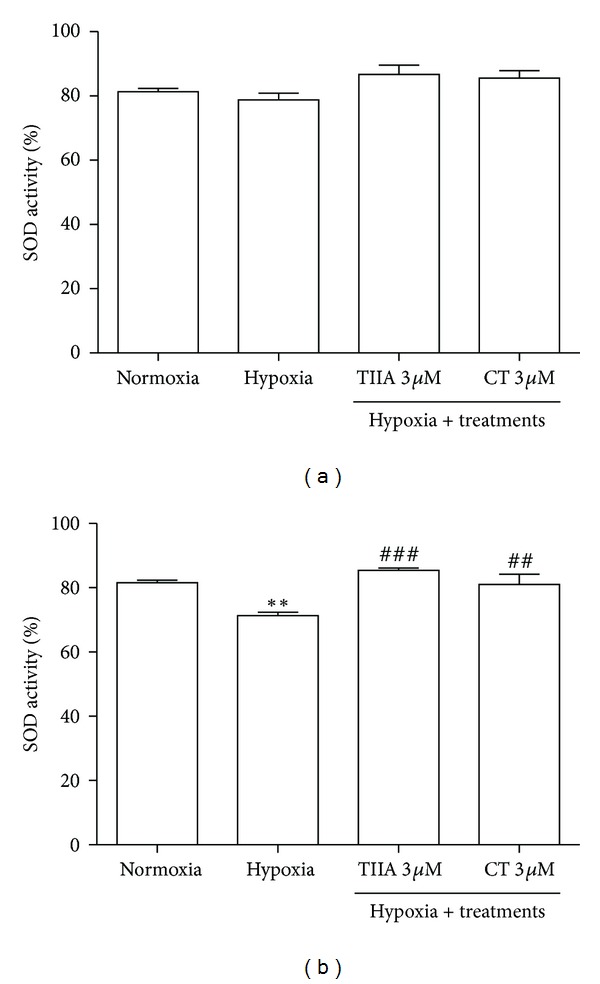
Effects of TIIA and CT on SOD enzyme activity. (a) Cytosolic SOD activity. (b) Mitochondrial SOD activity. Data shown are representative of four independent experiments. **P < 0.01  *versus normoxia*, ^##^ *P* < 0.01  *versus hypoxia,* and ^###^ *P* < 0.001  *versus hypoxia. *

Cytosolic SOD activity in hypoxia group (78.8 ± 4.1%) did not significantly (*P* > 0.05) change compared to normoxia control (81.3 ± 2.1%). In contrast, the cells in hypoxia showed a significant decrease in mitochondrial SOD activity by 12.6% compared to normoxia control. In the presence of TIIA and CT (3 *μ*M), the cytosolic SOD activity did not significantly change compared to hypoxia control. The decrease in mitochondrial SOD activity by hypoxia was restored when the cells were pretreated with 3 *μ*M TIIA or 3 *μ*M CT. There was no statistically significant difference in mitochondrial SOD activity between tanshinones treated groups and normoxia control (Figures 5(a) and 5(b)). 

### 3.6. Effects of TIIA and CT on Hypoxia-Induced Increase in Intracellular Nitric Oxide Production

**Figure 6:**
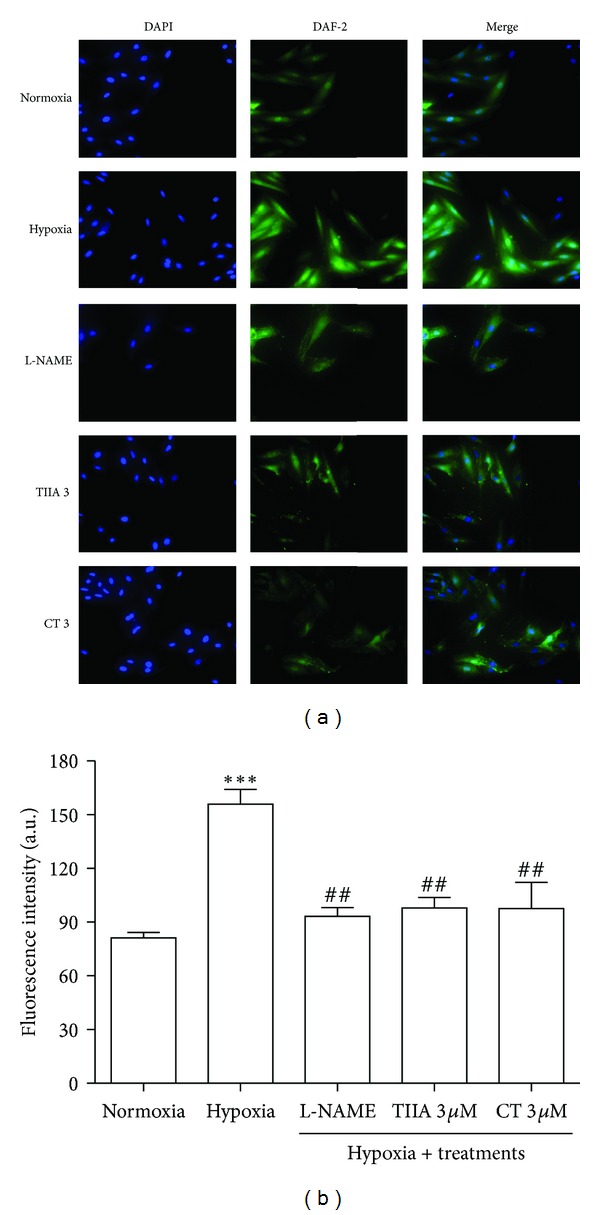
Effects of TIIA and CT on hypoxia-induced increase in intracellular nitric oxide production. (a) Cell images illustrate DAF-2 (green) and DAPI (blue) in each group. (b) Quantitative DAF-2 fluorescence intensity. Represented data are mean value of 500 each cells with 3 independent experiments (*n* = 3). ^##^ *P* < 0.01  *versus hypoxia*,  ***P < 0.001  *versus normoxia. *

Figure 6(a) illustrates the fluorescence images of cells stained with DAF-2. When compared with normoxia control, distinct intensification in DAF-2 fluorescence was observed in hypoxia group. The quantitative values of the florescence intensity of images were presented in Figure 6(b). Pretreatment with TIIA and CT significantly decreased the intracellular NO production. There was no statistical difference between tanshinones-treated groups and normoxia control. L-NAME (1 mM, NOS inhibitor) significantly (*P* < 0.01) reduced the intracellular NO production compared to hypoxia control.

### 3.7. Effects of TIIA and CT on Hypoxia-Induced Increase in Intracellular Ca^2+^ Level

**Figure 7:**
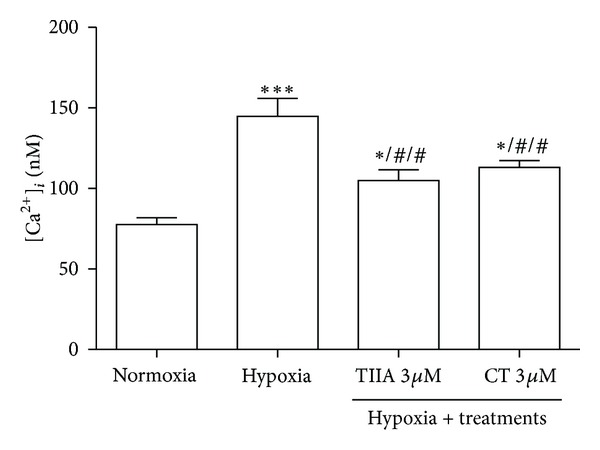
Effects of TIIA and CT on hypoxia-induced increase in intracellular calcium level. Data shown are representative of four independent experiments. *P < 0.05*  versus normoxia*, ^##^ *P* < 0.01  *versus hypoxia*, and ***P < 0.001  *versus normoxia. *

Intracellular Ca^2+^ ([Ca^2+^]_*i*_) level was significantly elevated in hypoxia group (*P* < 0.001) compared to normoxia control. Pretreatment with 3 *μ*M of TIIA and CT significantly (*P* < 0.01) prevented the [Ca^2+^]_*i*_ elevation compared to hypoxia control (Figure 7).

## 4. Discussion

The main finding of the present study is that TIIA and CT protect against chronic hypoxia-induced H9c2 cells injury by restoring cellular ATP contents, decreasing mitochondrial superoxide, intracellular NO, and calcium levels in H9c2 cells. This is consistent with previous observations that hypoxia-induced apoptosis was associated with ROS, NO, and calcium in myocardial cells [5, 25]. Additionally, cellular ATP contents, NO, and calcium are closely associated in mitochondrial ROS production and this suggests that chronic hypoxia-induced cell damages are related to mitochondrial dysfunction. 

Myocardial hypoxia is a main cause of cardiac dysfunction due to its triggering cell injury, apoptosis, and/or necrosis [1, 26]. The present study showed that the main cause of cell injury or death under the chronic hypoxia condition was associated with mitochondrial dysfunction with accompanying LDH release and cellular ATP depletion, which is consistent with previous reports [5, 27]. The protective actions of tanshinones against chronic hypoxia-induced cell injury indicate that these compounds may conserve mitochondrial function. Previous studies have reported cardioprotective effects of TIIA on H_2_O_2_-induced cell injury [28] and doxorubicin-induced cell apoptosis [29] in neonatal cardiomyocytes by protecting DNA integrity mitochondrial proteins and reducing intercellular ROS production. Similarly, the antiapoptotic effect of CT has been shown previously with actions of preventing mitochondrial-dependent apoptosis in nitric oxide induced neuroblastoma cells apoptosis [30]. The increase of ATP level by TIIA may be related to its protection of mitochondrial electron transport chain (ETC) function as ATP is mostly generated by oxidative phosphorylation, a process translocating protons by complex I/III/IV and subsequently uptake of the protons by ATP synthase accompanying the synthesis of ATP, in mitochondria ETC [31]. The finding that CT was less effective than TIIA in restoring cellular ATP contents may be related to a previous observation that CT enhanced AMP-activated protein kinase (AMPK) [32], as it has been known that AMPK is associated with energy homeostasis, mitochondrial function, and cell survival [33]. It will be interesting to investigate further the effects of tanshinones on AMPK activity in chronic hypoxia condition.

Previous studies on hypoxia-induced ROS generation have shown conflicting results. This could be due to a confusion of ROS examined (cytosolic and mitochondria). It has been shown that hypoxia decreased cytosolic superoxide generation but increased mitochondrial superoxide generation [34]. Consistent with this, a significant decrease in cytosolic superoxide generation, but increase in mitochondrial superoxide generation after hypoxia, was observed in the present study. Decreased cytosolic superoxide generation may be associated with lower oxygen level during hypoxia condition and/or decreased NADPH oxidase activity [2, 21]. Interestingly, the activity of cytosolic antioxidant enzyme superoxide dismutase was not significantly changed after hypoxia, indicating that this cytosolic antioxidant enzyme may not play a major role in cell injury and death pathway during chronic hypoxia. On the other hand, there was a significant increase of intracellular hydrogen peroxide/peroxynitrite production, as indicated by DCFH-DA fluorescence probe, suggesting that a mitochondrial-derived ROS component may be involved as shown by the effects of complex I and III inhibitors (rotenone and antimycin A). 

The present result is in line with a previous report showing that increased NO and ONOO^−^ generations resulted in enhanced mitochondrial superoxide generation by blocking mitochondrial electron transport chain [35]. NO can act as a physiological regulator of respiration by reversibly inhibiting cytochrome c-oxidase at the low concentrations (nanomolar). However, at higher concentrations NO can oxidize ubiquinol of ubiquinol-cytochrome c-reductase (Complex III) to increase unstable ubisemiquinone, which produces superoxide by univalent electron transfer to O_2_ [36]. Additionally, exposure to higher concentrations of NO can increase peroxynitrite formation which causes an inhibition of mitochondrial respiration at multiple sites (complex I, complex II, cytochrome c oxidase, the ATP synthase, aconitase, MnSOD, and creatine kinase) [37]. This implies that restoring electron transport chain function by reducing NO production, in addition to antioxidant enzyme activity, may help to reduce mitochondrial superoxide production during chronic hypoxia condition.

Since cytosolic ROS may not play major role in hypoxia-induced cell damages, it is not surprising to observe the lack of effect of TIIA and CT in intracellular ROS and NADPH oxidase activity. The important finding in this study is that the mitochondria superoxide generation was increased by hypoxia. This increase was significantly inhibited by TIIA and CT treatments, indicating that mitochondrial ROS plays a major role in cell damage-induced hypoxia. Interestingly, NO synthase inhibitor L-NAME also significantly inhibited mitochondrial superoxide generation, which suggests that endogenous NO may regulate the ROS production in hypoxia condition. It is possible that ROS may be generated from mitochondrial nitric oxide syntheses which may be uncoupled under hypoxic condition [38]. The observation of increase in mitochondrial superoxide dismutase activity and decrease in intracellular NO level by TIIA and CT in the present study is consistent with previous studies showing actions of tanshinones on regulating NO level and SOD activity in H_2_O_2_-induced cell injury and inflammation-induced cell death in endothelial cells [16, 39]. 

Interestingly, TIIA and CT significantly decreased intracellular NO and mitochondrial superoxide generations, but not peroxynitrite/hydrogen peroxide levels. Previous studies using the same DCF-DH probe found that TIIA significantly inhibited ROS generation induced by doxorubicin [14, 15]. However, it is not clear if the ROS in those studies is peroxynitrite/hydrogen peroxide specific as no specific inhibitors were used to validate the species of ROS observed. One possible explanation is that ROS labelled with DCF-DH may mainly be peroxynitrite as specific peroxynitrite inhibitor MnTBAP markedly reduced ROS generation (about 70%) in the present study. Thus, TIIA and CT may have a capacity to direct ROS production from peroxynitrite to hydrogen peroxide, as both compounds showed no significant effects on peroxynitrite/hydrogen peroxide production; even they significantly reduced NO and superoxide productions which theoretically should reduce peroxynitrite formation. Partial supporting evidence is that TIIA and CT increased mitochondrial SOD activity, which may result in increase in hydrogen peroxide formation. Further study is required to confirm this hypothesis. The effects of tanshinones on other antioxidant enzymes such as glutathione peroxidase and catalyse were not examined in this study which also requires further investigation. 

Changes in intracellular calcium level during hypoxia are important in mitochondrial functions, especially in mitochondrial membrane permeability transition pore opening [40]. Decreased intracellular ATP by hypoxia can decrease cellular pH by glycolysis activation and this elicits imbalancing in intracellular ion exchange and, subsequently, increases in intracellular calcium level [41]. This is consistent with the present finding showing that hypoxia-induced ATP depletion was accompanied with increased intracellular calcium level. The finding of inhibition of intracellular calcium by TIIA and CT is consistent with previous reports in neonatal cardiomyocytes and rat coronary artery [42, 43]. The increased intracellular calcium level is likely due to ATP depletion caused by hypoxia. Thus, it is possible that TIIA may regulate intracellular calcium through affecting ATP level. However, CT reduced intracellular calcium without affecting ATP levels. This suggests that other mechanisms such as endoplasmic reticulum-related stress, which also regulate intracellular calcium production [44], may also be involved. Additionally, studies have shown that increased intracellular calcium may increase mitochondrial ROS production [45, 46]. Therefore, tanshinones may have multiple targets of reducing mitochondrial ROS production.

In summary, the findings from the present study indicate that TIIA and CT protect H9c2 cells via preserving mitochondria function by reducing excess production of mitochondrial superoxide, SOD activity, intracellular NO, and calcium levels and restoring cellular ATP contents. These molecular mechanisms may be involved in the cardioprotective actions of TIIA and CT in hypoxic injuries. 

## References

[1] Cassavaugh J, Lounsbury KM (2011). Hypoxia-mediated biological control. *Journal of Cellular Biochemistry*.

[2] Santos CXC, Anilkumar N, Zhang M, Brewer AC, Shah AM (2011). Redox signaling in cardiac myocytes. *Free Radical Biology and Medicine*.

[3] Solaini G, Baracca A, Lenaz G, Sgarbi G (2010). Hypoxia and mitochondrial oxidative metabolism. *Biochimica et Biophysica Acta*.

[4] Gao Q, Wolin MS (2008). Effects of hypoxia on relationships between cytosolic and mitochondrial NAD(P)H redox and superoxide generation in coronary arterial smooth muscle. *American Journal of Physiology*.

[5] Kolamunne RT, Clare M, Griffiths HR (2011). Mitochondrial superoxide anion radicals mediate induction of apoptosis in cardiac myoblasts exposed to chronic hypoxia. *Archives of Biochemistry and Biophysics*.

[6] Walford GA, Moussignac R, Scribner AW, Loscalzo J, Leopold JA (2004). Hypoxia potentiates nitric oxide-mediated apoptosis in endothelial cells via peroxynitrite-induced activation of mitochondria-dependent and -independent pathways. *Journal of Biological Chemistry*.

[7] Chen J-X, Meyrick B (2004). Hypoxia increases Hsp90 binding to eNOS via PI3K-Akt in porcine coronary artery endothelium. *Laboratory Investigation*.

[8] Zhang DX, Gutterman DD (2007). Mitochondrial reactive oxygen species-mediated signaling in endothelial cells. *American Journal of Physiology*.

[9] Dong X-B, Yang C-T, Zheng DD (2012). Inhibition of ROS-activated ERK1/2 pathway contributes to the protection of H2S against chemical hypoxia-induced injury in H9c2 cells. *Molecular and Cellular Biochemistry*.

[10] Chen HW, Chien CT, Yu SL, Lee Y, Chen W (2002). Cyclosporine A regulate oxidative stress-induced apoptosis in cardiomyocytes: mechanisms via ROS generation, iNOS and Hsp70. *British Journal of Pharmacology*.

[11] Wu B, Liu M, Zhang S (2007). Dan Shen agents for acute ischaemic stroke. *Cochrane Database of Systematic Reviews*.

[12] Zhou L, Zuo Z, Chow MSS (2005). Danshen: an overview of its chemistry, pharmacology, pharmacokinetics, and clinical use. *Journal of Clinical Pharmacology*.

[13] Han J-Y, Fan J-Y, Horie Y (2008). Ameliorating effects of compounds derived from Salvia miltiorrhiza root extract on microcirculatory disturbance and target organ injury by ischemia and reperfusion. *Pharmacology & Therapeutics*.

[14] Gao J, Yang G, Pi R (2008). Tanshinone IIA protects neonatal rat cardiomyocytes from adriamycin-induced apoptosis. *Translational Research*.

[15] Hong H-J, Liu J-C, Chen P-Y, Chen J-J, Chan P, Cheng T-H (2012). Tanshinone IIA prevents doxorubicin-induced cardiomyocyte apoptosis through Akt-dependent pathway. *International Journal of Cardiology*.

[16] Zhou Z, Wang S-Q, Liu Y, Miao A-D (2006). Cryptotanshinone inhibits endothelin-1 expression and stimulates nitric oxide production in human vascular endothelial cells. *Biochimica Et Biophysica Acta*.

[17] Pan C, Lou L, Huo Y (2011). Salvianolic acid B and Tanshinone IIA attenuate myocardial ischemia injury in mice by NO production through multiple pathways. *Therapeutic Advances in Cardiovascular Disease*.

[18] Kim MJ, Moon C-H, Kim M-Y (2005). KR-32570, a novel Na+ / H+ exchanger-1 inhibitor, attenuates hypoxia-induced cell death through inhibition of intracellular Ca^2+^ overload and mitochondrial death pathway in H9c2 cells. *European Journal of Pharmacology*.

[19] Rakhit RD, Edwards RJ, Mockridge JW (2000). Nitric oxide-induced cardioprotection in cultured rat ventricular myocytes. *American Journal of Physiology*.

[20] Griendling KK, Minieri CA, Ollerenshaw JD, Alexander RW (1994). Angiotensin II stimulates NADH and NADPH oxidase activity in cultured vascular smooth muscle cells. *Circulation Research*.

[21] Wu W, Platoshyn O, Firth AL, Yuan JX-J (2007). Hypoxia divergently regulates production of reactive oxygen species in human pulmonary and coronary artery smooth muscle cells. *American Journal of Physiology*.

[22] Chen H-W, Chien C-T, Yu S-L, Lee Y-T, Chen W-J (2002). Cyclosporine A regulate oxidative stress-induced apoptosis in cardiomyocytes: mechanisms via ROS generation, iNOS and Hsp70. *British Journal of Pharmacology*.

[23] McConkey DJ, Nutt L (2004). Measurement of changes in intracellular calcium during apoptosis. *Methods in Molecular Biology*.

[24] Grynkiewicz G, Poenie M, Tsien RY (1985). A new generation of Ca^2+^ indicators with greatly improved fluorescence properties. *Journal of Biological Chemistry*.

[25] Patterson AJ, Xiao D, Xiong F, Dixon B, Zhang L (2012). Hypoxia-derived oxidative stress mediates epigenetic repression of PKC*ε* gene in foetal rat hearts. *Cardiovascular Research*.

[26] Jung F, Weiland U, Johns RA, Ihling C, Dimmeler S (2001). Chronic hypoxia induces apoptosis in cardiac myocytes: a possible role for Bcl-2-like Proteins. *Biochemical and Biophysical Research Communications*.

[27] Kim MJ, Moon C, Kim MH, Lee SH, Baik EJ, Jung Y (2004). Role of PKC-*δ* during hypoxia in heart-derived H9c2 cells. *Japanese Journal of Physiology*.

[28] Fu J, Huang H, Liu J, Pi R, Chen J, Liu P (2007). Tanshinone IIA protects cardiac myocytes against oxidative stress-triggered damage and apoptosis. *European Journal of Pharmacology*.

[29] Gao J, Yang G, Pi R (2008). Tanshinone IIA protects neonatal rat cardiomyocytes from adriamycin-induced apoptosis. *Translational Research*.

[30] Mahesh R, Jung HW, Kim GW, Kim YS, Park Y-K (2012). Cryptotanshinone from salviae miltiorrhizae radix inhibits sodium-nitroprusside-induced apoptosis in neuro-2a cells. *Phytotherapy Research*.

[31] Kadenbach B (2003). Intrinsic and extrinsic uncoupling of oxidative phosphorylation. *Biochimica et Biophysica Acta*.

[32] Eun JK, Jung S, Kun HS (2007). Antidiabetes and antiobesity effect of cryptotanshinone via activation of AMP-activated protein kinase. *Molecular Pharmacology*.

[33] Terai K, Hiramoto Y, Masaki M (2005). AMP-activated protein kinase protects cardiomyocytes against hypoxic injury through attenuation of endoplasmic reticulum stress. *Molecular and Cellular Biology*.

[34] Gao Q, Wolin MS (2008). Effects of hypoxia on relationships between cytosolic and mitochondrial NAD(P)H redox and superoxide generation in coronary arterial smooth muscle. *American Journal of Physiology*.

[35] Ilangovan G, Osinbowale S, Bratasz A (2004). Heat shock regulates the respiration of cardiac H9c2 cells through upregulation of nitric oxide synthase. *American Journal of Physiology*.

[36] Jones CI, Han Z, Presley T (2008). Endothelial cell respiration is affected by the oxygen tension during shear exposure: role of mitochondrial peroxynitrite. *American Journal of Physiology*.

[37] Brown GC, Borutaite V (2002). Nitric oxide, mitochondria, and cell death. *IUBMB Life*.

[38] Verhaar MC, Westerweel PE, van Zonneveld AJ, Rabelink TJ (2004). Free radical production by dysfunctional eNOS. *Heart*.

[39] Lin R, Wang W-R, Liu J-T, Yang G-D, Han C-J (2006). Protective effect of tanshinone IIA on human umbilical vein endothelial cell injured by hydrogen peroxide and its mechanism. *Journal of Ethnopharmacology*.

[40] Halestrap AP, Pasdois P (2009). The role of the mitochondrial permeability transition pore in heart disease. *Biochimica et Biophysica Acta*.

[41] Brookes PS, Yoon Y, Robotham JL, Anders MW, Sheu S (2004). Calcium, ATP, and ROS: a mitochondrial love-hate triangle. *American Journal of Physiology*.

[42] Yang P, Jia Y-H, Li J, Li L-J, Zhou F-H (2010). Study of anti-myocardial cell oxidative stress action and effect of tanshinone IIA on prohibitin expression. *Journal of Traditional Chinese Medicine*.

[43] Lam FFY, Yeung JHK, Chan KM, Penelope MYO (2008). Mechanisms of the dilator action of cryptotanshinone on rat coronary artery. *European Journal of Pharmacology*.

[44] Park IJ, Kim MJ, Park OJ (2012). Cryptotanshinone induces ER stress-mediated apoptosis in HepG2 and MCF7 cells. *Apoptosis*.

[45] Kaminishi T, Kako KJ (1989). Sensitivity to oxidants of mitochondrial and sarcoplasmic reticular calcium uptake in saponin-treated cardiac myocytes. *Basic Research in Cardiology*.

[46] Sun H-Y, Wang N-P, Kerendi F (2005). Hypoxic postconditioning reduces cardiomyocyte loss by inhibiting ROS generation and intracellular Ca^2+^ overload. *American Journal of Physiology*.

